# Supplementary figures and images for: Effects of the menstrual cycle phase on anterior cruciate ligament neuromuscular and biomechanical injury risk surrogates in eumenorrheic and naturally menstruating women: A systematic review
Source: PLoS One. 2023 Jan 26;18(1):e0280800. doi: 10.1371/journal.pone.0280800 (PMC9879429; doi:10.1371/journal.pone.0280800)

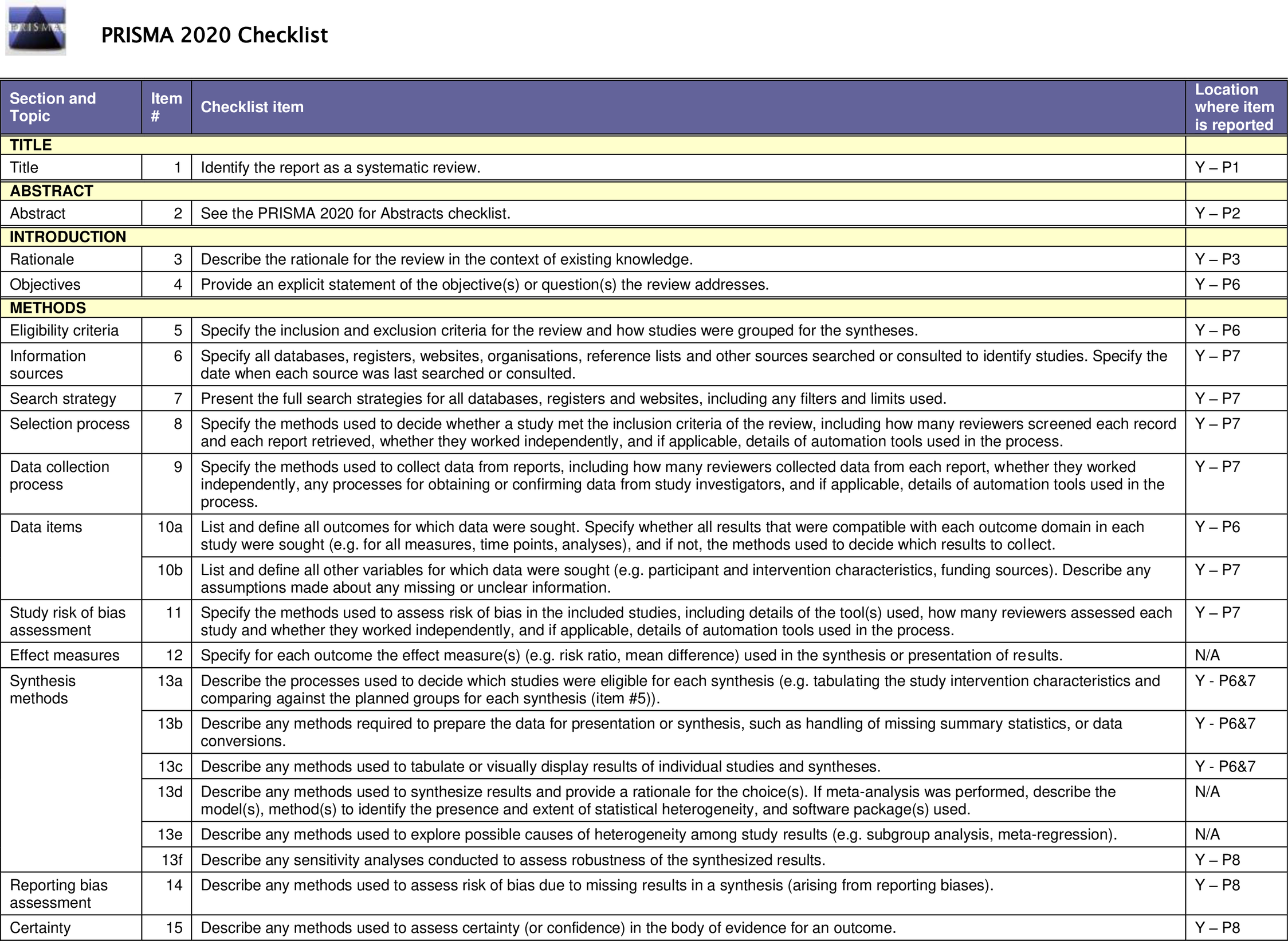

Supplement: S1 Checklist — (TIF) [file pone.0280800.s001.tif]
