# Supplementary material for: Effects of the menstrual cycle phase on anterior cruciate ligament neuromuscular and biomechanical injury risk surrogates in eumenorrheic and naturally menstruating women: A systematic review
Source: PLoS One. 2023 Jan 26;18(1):e0280800. doi: 10.1371/journal.pone.0280800 (PMC9879429; doi:10.1371/journal.pone.0280800)
Supplement: Attachment — Submitted filename: Response to Reviewers.docx [file pone.0280800.s002.docx]

Reviewer #1: In the manuscript ‘’Effects of The Menstrual Cycle Phase on Anterior Cruciate Ligament Neuromuscular and Biomechanical Injury Risk Surrogates in Eumenorrheic and Naturally Menstruating Women: A Systematic Review and Recommendations for Future Research’’, the authors attempted to address whether the menstrual cycle has an impact on the neuromuscular and biomechanical risk surrogates of ACL injury patients. Furthermore, the authors also provided comments on the current literature and propose suggestions for future research on the menstrual cycle and ACL injuries.

Although the manuscript may be of interest for the readers of PLOS ONE, it suffers some important issues.

General comments:
The manuscript is written with a high standard of English language. This promotes good readability and a general flow. Moreover, the article adheres to appropriate reporting guidelines for the current systematic review as it was conducted according to the PRISMA protocol. However, the manuscript in its current form is too long and there are some concerns with regards to reporting of the results.

Response: Thank you for your comment. We have done our best to reduce the word count, reducing the word count overall from 6777 words to 5728. This has included some extra additions in the methods and discussion as your requests and reviewer 2.

Specific comments:
Title page
1. The title of the article is somewhat long, perhaps it would be useful to shorten it, for example: ‘’Effects of the menstrual cycle phase on anterior cruciate ligament injury risk surrogates in menstruating women: a systematic review’’.

Response: Thank you for your comment. We agree that it is slightly long; however, we believe it is more appropriate and important to use the scientific terminology as recommended by Elliott-Sale et al. (2021) to specifically provide context regarding the reproductive profiles of the women of this systematic review (i.e., Eumenorrheic and Naturally Menstruating). Moreover, there are a plethora of different ACL risk factors and surrogates; however, we are reluctant to remove the terms “neuromuscular and biomechanical” because we feel the inclusion of there terms provides more context and scope for the systematic review. However, in light of your comment and R2’s, we have removed the term “and recommendations for future research” from the title.
2. Line 20: please change ‘’1 figures’’ to ‘’1 figure’’.

Response: Thank you for your comment. This has been amended.

Abstract
1. Lines 43-48, it would be helpful to add which quality assessment tool(s) was used for the included studies of the systematic review. This could also be added to the last sentence of the results section (line 53) where the overall study quality of included studies is presented.

Response: Thank you for your comment. This has been amended. Please see line 50-53 and 58.

2. In the background, only the primary aim of the study is provided while in the conclusion, both the primary and secondary aims are listed. The background and the conclusion section should match in the abstract.

Response: Thank you for your comment. This has been amended where we have added a brief indication of the secondary aim. Please see line 42.

Introduction
1. The first, second, third and fourth paragraph of the introduction are some general aspects of ACL injuries which are already well known. These paragraphs may perhaps be removed from the manuscript so that the focus lies on the menstrual cycle and its relationship to ACL injuries.

Response: Thank you for your comment. We have reduced the introduction by 2 paragraphs / 451 words (1780 to 1329 words) and we hope that the introduction is more concise. We decided that the content related to the biomechanical and neuromuscular risk factors should stay, given the importance of these risk factors on potential mechanical loads and the fact that these risk factors were the inclusion criteria for the systematic review, thus providing context for the reader.

2. Lines 171-75, the primary objective of the manuscript is provided twice although in different forms. I would suggest that the question ‘’does the way women perform the aforementioned high-risk manoeuvres predispose them to potentially greater non-contact ACL injury risk based on neuromuscular and biomechanical surrogates of non-contact ACL injury risk at a particular MC stage?’’ is removed from this paragraph.

Response: Thank you for your comment. This has been amended and we have removed your suggested sentence. Please see line xx.

Methods
1. Line 179 – the study protocol was not pre-registered, however – were the review methods established prior to the conduct of the review? Clarifying this to the reader would be helpful.

Response: Thank you for your comment. This has been amended and we have added the following: however, the review methods were established prior to conducting the review. Please see line 153.

2. Table 1. The authors use references in the PICOS section, which in my opinion is not necessary. In the last row, the authors provide the data that was extracted. Was the data extraction process performed in duplicate?

Response: Thank you for your comment. With respect, would still like to include the references in table 1. We feel the inclusion of references further supports and justifies the PICOS strategy for specific population, method, comparator, or outcome criteria. Yes, performed in duplicate. This is added on Line 182.
3. Line 230 – it is stated that all studies in this review were observational although in Table 1 ‘’study design’’ only experimental studies were included. I believe that these 2 study designs are not equivalent to each other as observational studies can for instance be cohort studies while experimental studies can be performed in laboratories.

Response: Thank you for your comment. Apologies for this error. This has been amended.
Results
1. Line 243 – it appears as if the table numbers have been written in the wrong order as table 2 includes the quality assessment according to Downs and Black checklist and not the study characteristics. Please revise the order of the tables. Additionally, it is stated that the sample sizes ranged from 10-28 although one study (Shultz et al.) had a sample size of 71 which leads to a range of 10-71.

Response: Thank you for your comment. Apologies, this has been amended. Please see line 225-228.

2. Line 290-297 – the a-priori quality should be provided first, followed by the revised quality scores/quality evidence, i.e. the Downs and Black checklist scores, followed by the GRADE.

Response: Thank you for your comment. This has been amended in light of your suggestions. Please see line 274-280.

Table 3
1. The table includes valuable information of the study designs but is somewhat big, perhaps divide this table into 2 separate ones?

Response: Thank you for your comment. While it is a quite a large table, we feel it is best to contain all of this information in one table so all information is collated in one area and table. If accepted, the journal publishers can also format the table to aid with clarity.

**Discussion**
1. The discussion in its current form is too long. For example, each paragraph includes detailed information on specific studies, perhaps these could be shortened?

Response: Thank you for your comment. We have done our best to reduce the word count of the discussion. As such, we have reduced the length by 742 words, from 2882 to 2140 words.

2. The discussion section refers to the included tables of the manuscript which should be removed, for instance see line 335.

Response: Thank you for your comment. References to tables have been removed in the discussion.

3. A comment on the revised Downs and Black checklist used in the current manuscript would be welcomed, is it validated for instance?

Response: Thank you for your comment. We have commented on this on line 455.
4. The discussion section is currently lacking a strength/limitation section of the methodology applied in the systematic review.

Response: Thank you for your comment. We have added a small paragraph regarding the strength and limitations. Please see line 454-459.

5. A short paragraph addressing the biochemical analyses and ovulation kits used to determine the phase of the menstrual cycle of included studies would be valuable to the manuscript.
Response: Thank you for your comment. We are conscious of the word count and are, with respect, reluctant to include further information. We do briefly discuss which studies used biochemical analysis / ovulation kits in the results section (line 249-251) and information is also presented in Table 4.
Author contributions
The author contributions are stated in the methods section as all in the author contribution section, perhaps these could be listed in only one paragraph?

Response: Thank you for your comment. Unfortunately, we do not fully understand this comment. We have followed the author contributions guidance as per PlosOne journal specifications.

Reviewer #2: Dear authors,
As a reviewer, I appreciate your scientific efforts in elaborating the topic of the submitted study. The topic of the study is interesting und not well studied so far. The reviewer believes that the submitted study is of interest to the readership of PLOS ONE. However, there are several limitations that should be addressed before the study can be considered for publication in PLOS ONE.

The main limitations / revisions needed are as follows:

-The clinical relevance of the study needs to be highlighted. Why is this study needed and how is this study useful in daily clinical practice / research.
- Data analysis is poorly described and quantitative data were rarely reported. This can be done better.
-The manuscript is well written. However, the manuscript is far too long and needs to be shortened.

Response: Thank you for your comments. In the introduction, we high highlighted the clinical relevance of this study. Please see lines 142-145 and 151-153. We have now added a data analysis collations section. Please see line 212-217. We have done our best to reduce the word count, reducing the word count overall from 6777 words to 5728. This has included some extra additions in the methods and discussion as your requests and reviewer 1. We have reduced the introduction by 2 paragraphs / 451 words (1780 to 1329 words) and we hope that the introduction is more concise. Additionally, we have reduced the length of the discussion by 742 words, from 2882 to 2140 words.
Title
The reviewer recommends to delete “and recommendations for future research”.

Response: Thank you for your comment. This has been amended.

Abstract
Background: Adequate.
Methods: Line 48: The reviewer recommends to add one sentence about data analysis (e.g., quantitative, qualitative etc.).

Response: Thank you for your comment. This has been amended. We have added the following on line 49: “Quantitative data pertaining to study methodology, participant, MC phase and verification, ACL injury risk surrogates, reliability measures, and results were obtained for qualitative analysis.”

Results: Line 53: Please be more specific. Provide quantitative data for “low to very low” study quality (i.e., modified Downs and Black checklist).

Response: Thank you for your comment. This has been amended. Please see line 58 where we state the following: “Study quality and quality of evidence was low to very low (7-9 Modified Downs and Black Checklist score 7-9; and GRADE).”

Conclusions: The reviewer recommends to shorten the Conclusion section of the abstract. In addition, add one sentence about the clinical relevance of this study.

Response: Thank you for your comment. Due to the extensive nature of this systematic review, and complexities and caveats with the evaluation of the literature, we believe is an inappropriate to try and summarise this in one sentence. Previous systematic reviews have used multiple sentences in the abstract conclusion when contextualising their findings. Please see McNulty et al. 2021 and Somerson et al. 2019 in our reference list. We have amended some sentences to further highlight the clinical relevance of our review findings. Please see line 60-66.

Level of evidence: Is missing, please add.

Response: Thank you for your comment. This has been added. Please see line 67.

Key Words: Adequate.

Introduction
Far too long. Please shorten and focus on the topic of the study. Why is this study needed? Highlight particular controversies in the introduction section. Adequate objectives. Hypothesis of the study is missing. Please add.

Response: Thank you for your comments. In the introduction, we high highlighted the clinical relevance of this study. Please see lines 142-145 and 151-153. We have reduced the introduction by 2 paragraphs / 451 words (1780 to 1329 words) and we hope that the introduction is more concise.

Hypotheses have also been added. Please see line 151: “It was hypothesised that differences in neuromuscular and biomechanical injury risk surrogates would be observed between MC phases in eumenorrheic and naturally menstruating women.”

Line 65, 121: Define abbreviations when they are used for the first time (i.e., ACL, MC).

Response: Thank you for your comment. This has been added. Please see line 69 and 96.

Material and Methods
There are several missing items in the material and methods section that should be added:
-Who performed data extraction?
-Which data were collected from each study for data synthesis?
-Statistical analysis and methods of data synthesis

Response: Thank you for your comment. We have now added a data collation section (2.4) which provides information pertaining to data extraction and specific data. No statistical analysis / meta-analysis was performed due to the heterogeneity in methodology and outcome measures.

Line 179-180: Why was this review not registered in advance? This is a major limitation and should be mentioned in the limitations section. This reduces the quality of the systematic review.

Response: Thank you for your comment. In hindsight, we should have registered the review. We have acknowledged this as a limitation. Please see line 460.

Line 187: It is recommended to add the full search strategy as a supplemental file.

Response: Thank you for your comment. The full search strategy is provided on line 173.

Line 219-225: This is somewhat confusing. Based on Table 1, only “studies that verified participants’ MC phase via biochemical analysis (i.e., blood/serum analysis) and / or ovulation kits were included, in line with McNulty et al.”. Therefore, no study should have been downgraded? Please clarify.

Response: Thank you for your comment. Yes, if they failed to do blood samples but used an ovulation kit, they were downgraded. Likewise, if they used a blood sample but no ovulation kits, then again, they were downgraded. Thus, to maintain their rating, they had to do both. This was the case for study 1, 2, 3, and 7 which were downgraded.

Results
In general, it would be good to present a quantitative data synthesis. However, the studies may have been so heterogeneous that data synthesis was not possible, which is a limitation of the study. Please comment.

Response: Thank you for your comment. Yes, we have now included a data collation section (2.4) and have acknowledged that that the data and methodologies were heterogeneous which precluded a meta-analysis. Please see line 281 and 460.

Discussion
The discussion section is well written. However, the discussion section is far too long. Please try to shorten. Focus on the topic of the study and highlight the clinical relevance of this study. How can the results of this study be useful in daily clinical practice?

Response: Thank you for your comment. We have reduced the length of the discussion by 742 words, from 2882 to 2140 words. We have further highlighted the clinical relevance in certain sections. Please see lines 345-347, 398, 439-442. However, because of the low quality of evidence and methodological quality, this review is primarily highlighting the future directions of research and methodological approaches to improve research quality and data to increase our understanding of the menstrual cycle on ACL injury risk.


Line 329: It is recommended to start the discussion section as follows: “The most important finding of this study was …”

Response: Thank you for your comment. This has been amended. Please see line 305.

Conclusion
Far too long. Please shorten. In the conclusion section, the authors should “conclude” their findings in 2-5 sentences. In addition, the clinical relevance of the study should be highlighted. Moreover, it is recommended to delete quotations (both for references and tables) in the conclusion section.

Response: Thank you for your comment. We have reduced the length from 489 to 277 words and have removed quotations and references.

Figures
Figure 1: Adequate.

Tables
Table 1: How did the authors confirm that the women were eumenorrheic? Please clarify.

This was based on the methodologies reported by the research group and interpreted according to the definition presented by Elliott-Sale et al. 2021 for eumenorrheic classification.

Table 1: Please be more specific and define biomechanical and/or neuromuscular surrogates.

Response: Thank you for your comment. This has been added.
Table 2: Adequate.
Table 3: Adequate.
Table 4 + 5: Exhaustive and busy table. Try to shorten.
Response: Thank you for your comment. We feel these are critical tables for the systematic review and contain relevant and highly important information. In particular, Table 5 is central for highlighting the limitations and recommended areas for future research to improve research quality in this topic area. As such, with respect, we would like to keep the tables as they are.
References
Adequate.
