# Supplementary material for: Effects of the menstrual cycle phase on anterior cruciate ligament neuromuscular and biomechanical injury risk surrogates in eumenorrheic and naturally menstruating women: A systematic review
Source: PLoS One. 2023 Jan 26;18(1):e0280800. doi: 10.1371/journal.pone.0280800 (PMC9879429; doi:10.1371/journal.pone.0280800)
Supplement: Attachment — Submitted filename: Re-revision Horvath.docx [file pone.0280800.s003.docx]

**Re-review of revised manuscript ‘’Effects of The Menstrual Cycle Phase on Anterior Cruciate Ligament Neuromuscular and Biomechanical Injury Risk Surrogates in Eumenorrheic and Naturally Menstruating Women: A Systematic Review and Recommendations for Future Research’’, Dos’Santos et al.**

**Reviewer: Alexandra Horvath, PONE-D-22-07730R1**

**Abstract:**

On line 59, please change (7-9 Modified Downs and Black Checklist score 7-9; and GRADE) so that it is understandable which score corresponds to which quality assessment/grading tool.

Line 65-66 – remove this sentence as it does not add additional value to the conclusion section.

**Methods:**

In table I ‘’study design’’, please change experimental to clinical or human.

Who performed the literature search in PubMed, Medline (OVID), SPORTDiscus, and Web of Science databases?

Line 169, please add the reference to the guideline that was used.

**Results**

I would recommend that ‘’statistically’’ is removed from ‘’no statistically significant’’ on line 255. Furthermore, what does ‘’meaningful difference’’ refer to on line 255?

**Discussion**

The sentence on line 305-308 is very long, I would suggest that the sentence is divided into two.

Please remove reference to table on line 454.

Line 461 – please add which type of bias(es) the review was subjected to.

**Conclusion**

The conclusion section is far too long. It needs to be shortened.
