# Supplementary material for: Effects of the menstrual cycle phase on anterior cruciate ligament neuromuscular and biomechanical injury risk surrogates in eumenorrheic and naturally menstruating women: A systematic review
Source: PLoS One. 2023 Jan 26;18(1):e0280800. doi: 10.1371/journal.pone.0280800 (PMC9879429; doi:10.1371/journal.pone.0280800)
Supplement: Attachment — Submitted filename: Response to Reviewers.docx [file pone.0280800.s004.docx]

**Re-review of revised manuscript ‘’Effects of The Menstrual Cycle Phase on Anterior Cruciate Ligament Neuromuscular and Biomechanical Injury Risk Surrogates in Eumenorrheic and Naturally Menstruating Women: A Systematic Review and Recommendations for Future Research’’, Dos’Santos et al.**

**Reviewer: Alexandra Horvath, PONE-D-22-07730R1**

**Abstract:**

On line 59, please change (7-9 Modified Downs and Black Checklist score 7-9; and GRADE) so that it is understandable which score corresponds to which quality assessment/grading tool.

Response: Thank you for your comment. In a previous round of revisions, we were told to include this. Both of these tools use descriptors to classify the evidence as low or very low.

Line 65-66 – remove this sentence as it does not add additional value to the conclusion section.

Response: Thank you for your comment. This has been removed.

**Methods:**

In table I ‘’study design’’, please change experimental to clinical or human.

Response: Thank you for your comment. This has been amended.

Who performed the literature search in PubMed, Medline (OVID), SPORTDiscus, and Web of Science databases?

Response: Thank you for your comment. This has been added. Please see line 117.

Line 169, please add the reference to the guideline that was used.

Response: Thank you for your comment. This has been added. Please see line 119.

**Results**

I would recommend that ‘’statistically’’ is removed from ‘’no statistically significant’’ on line 255. Furthermore, what does ‘’meaningful difference’’ refer to on line 255?

Response: Thank you for your comment. This has been removed throughout the document. Please see line section 3. Meaningful is a term to denote inferences regarding the magnitude of differences, as suggested by Will Hopkins <https://www.sportsci.org/jour/05/ambwgh.htm> For instance, there may indeed be significant differences observed; however, the magnitude of the differences (i.e., effect size) may be trivial / small and thus not practically or clinically meaningful. Conversely, you may observe no significant differences; however, a practically or clinically meaningful effect size could be observed. As such, we would like to keep the term “meaningful” in the manuscript.

**Discussion**

The sentence on line 305-308 is very long, I would suggest that the sentence is divided into two.

Response: Thank you for your comment. This has been amended to two sentences.

Please remove reference to table on line 454.

Response: Thank you for your comment. This has been removed.

Line 461 – please add which type of bias(es) the review was subjected to.

Response: Thank you for your comment. This has been added. Please see line 354-356.

**Conclusion**

The conclusion section is far too long. It needs to be shortened.

Response: Thank you for your comment. This has been reduced from 277 to 186 words

Reviewer #2: Dear authors,
The first revision substantially improved the quality of the study. However, the biggest limitation of the manuscript is that it is still far too long. In particular, the introduction, discussion, and conclusion sections need to be shortened. The authors should focus on the most important and at the same time clinically relevant topics. A better structure and shorter version would significantly improve the study and maintain the excitement of reading throughout the entire manuscript. The reviewer recommends that the aforementioned limitations be addressed before the study can be considered for publication in PLOS ONE.
See the detailed comments on each section below.

Title
Much better now. Adequate title.

Response: Thank you for your comment.

Abstract
According to the submission guidelines ([https://journals.plos.org/plosone/s/submission-guidelines](https://protect-eu.mimecast.com/s/7ScWCLY9XIXZmBXSjbVwb?domain=journals.plos.org)), the abstract should not exceed 300 words. In the current version, the abstract has more than 400 words. Please shorten the abstract and focus on the most important information.
Response: Thank you for your comment. This has been reduced from 402 to 300 words.

Background: Adequate.
Methods: Line 50-53: In order to shorten the abstract, the authors consider to delete this information from the abstract. However, this information is of utmost importance in the main manuscript.

Response: Thank you for your comment and suggestion. This has been amended.

Results: Adequate.

Response: Thank you for your comment.

Conclusions: Despite the response of the authors, the conclusion is still too long. Try to shorten.
Response: Thank you for your comment. This has been reduced from 90 to 74 words.

Introduction
The introduction is far too long. This was already mentioned by both reviewers in the first review. Try to shorten the introduction to no more than 500 words.

Response: Thank you for your comment. We have amended the introduction, removing two main paragraphs and therefore have 4 short paragraphs. As such, the word count is now 691 words, having previously been 1329 words. We have done our best to remove all unnecessary information but feel the remaining content must remain in the introduction to provide an appropriate background and context. Overall, the word count is quite short for an introduction, and the total word count is 4102 words which I am sure you agree is low for a systematic review.

Line 69-86: The information in the first paragraph is well known. The authors should summarize the most important information regarding the submitted systematic review in 1-2 sentences or consider deleting the first paragraph.

Response: Thank you for your comment. We have deleted this first paragraph.
Line 87-98: This paragraph is a good introduction into the topic of the systematic review.

Response: Thank you for your comment. We have started from here. Please see line 61.
Line 109-111: Consider to delete this part.

Response: Thank you for your comment. We have deleted this.

Material and Methods
The authors did a good job on revising the materials and methods section. No further comments.
Response: Thank you for your comment.

Results
The authors may shorten the results section. Try to avoid repetitions between tables and written text.
Moreover, the authors did a good job on revising the results section. No more comments.
Response: Thank you for your comment. We have removed ~220 words, reducing the word count from 886 words to 664 words.

Discussion
The discussion section is still far too long. It is not intended to report all studies of the research topic and their results. Rather, a discussion / interpretation of the most important findings of the systematic review with already existing literature should be performed. Controversies should be discussed and clinically relevant results highlighted.
Response: Thank you for your comment. We have removed ~700 words from the discussion, reducing the word count 2142 words to 1375 words, leaving the most clinically relevant results and applications.
Conclusion
The conclusion section is still too long. The authors should shorten the conclusion and focus on the most significant and clinically relevant findings. Line 480-486: The reviewer recommends to move this part to the discussion section.

Response: Thank you for your comment. This has been reduced from 277 to 185 words.
