# Supplementary material for: Effects of the menstrual cycle phase on anterior cruciate ligament neuromuscular and biomechanical injury risk surrogates in eumenorrheic and naturally menstruating women: A systematic review
Source: PLoS One. 2023 Jan 26;18(1):e0280800. doi: 10.1371/journal.pone.0280800 (PMC9879429; doi:10.1371/journal.pone.0280800)
Supplement: Attachment — Submitted filename: Re-revision Horvath .docx [file pone.0280800.s005.docx]

**Re-revision of revised manuscript ‘’Effects of The Menstrual Cycle Phase on Anterior Cruciate Ligament Neuromuscular and Biomechanical Injury Risk Surrogates in Eumenorrheic and Naturally Menstruating Women: A Systematic Review and Recommendations for Future Research’’, Dos’Santos et al.**

**Reviewer: Alexandra Horvath, PONE-D-22-07730R1**

I have no further comments to add.
